# Vulnerability to Meningococcal Disease in Immunodeficiency Due to a Novel Pathogenic Missense Variant in *NFKB1*

**DOI:** 10.3389/fimmu.2021.767188

**Published:** 2021-12-24

**Authors:** Manfred Anim, Georgios Sogkas, Gunnar Schmidt, Natalia Dubrowinskaja, Torsten Witte, Reinhold Ernst Schmidt, Faranaz Atschekzei

**Affiliations:** ^1^ Department of Rheumatology and Immunology, Hannover Medical School, Hannover, Germany; ^2^ Hannover Biomedical Research School (HBRS), Hannover Medical School, Hanover, Germany; ^3^ RESIST - Cluster of Excellence 2155 to Hanover Medical School, Satellite Center Freiburg, Hanover, Germany; ^4^ Department of Human Genetics, Hannover Medical School, Hannover, Germany

**Keywords:** common variable immune deficiency (CVID), NFKB1, Nfkb1 (p50), hypogammaglobulinemia, primary antibody deficiency (PAD)

## Abstract

NF-κB1 deficiency is suggested to be the most common cause of common variable immunodeficiency (CVID). *NFKB1* encodes for the p105 precursor protein of NF-κB1, which is converted into the active transcriptional subunit p50 through proteasomal processing of its C-terminal half upon stimulation and is implicated in the canonical NF-kB pathway. Rare monoallelic *NFKB1* variants have been shown to cause (haplo) insufficiency. Our report describes a novel *NFKB1* missense variant (c.691C>T, p.R230C; allele frequency 0.00004953) in a family vulnerable to meningitis, sepsis, and late-onset hypogammaglobulinemia. We investigated the pathogenic relevance of this variant by lymphocyte stimulation, immunophenotyping, overexpression study and immunoblotting. The ectopic expression of p50 for c.691 C>T restricted transcriptionally active p50 in the cytoplasm, and immunoblotting revealed reduced p105/50 expression. This study shows that the deleterious missense variant in NFKB1 adversely affects the transcriptional and translational activity of NFκB1, impairing its function. Patients immunological parameters show a progressive course of hypogammaglobulinemia, which may partially account for the incomplete disease penetrance and suggest the need for closer immunological monitoring of those mutation carriers.

## Introduction

Primary antibody deficiencies, particularly common variable immunodeficiency (CVID), is the most common symptomatic primary immunodeficiency disorder. Patients with CVID have a highly variable clinical presentation. Besides an increased susceptibility to upper and/or lower respiratory infections, patients also present a high incidence of severe bacterial infections such as sepsis and meningitis and immune dysregulation features including lymphoproliferative, gastrointestinal and autoimmune manifestations (1–3). Before diagnosing CVID, secondary cause of antibody deficiency (SAD) must be excluded in clinical practice (4). So far, several monogenic defects such as *ICOS, CD19, CD20, CD21, CD27, CD81, IL21, IL21R, LRBA, PRKCD, RAC2, TNFSF12, CTLA4, PLCG2, NFKB1, NFKB2, PIK3CD, PIK3R1, VAV1, BLK, IKZF1, IRF2BP2*, as well as mutations in *TNFRSF13B* and *TNFRSF13C* have been identified in CVID (1, 5, 6). Recently NF-κB1 haplo (insufficiency) has been described as a novel monogenic cause of CVID (6). Several independent studies have reported that loss-of-function variants in *NFKB1* are probably the common cause of antibody deficiency with a highly variable clinical and immunological presentation (7). The nuclear factor of kappa light polypeptide gene enhancer in B cells (NF-κB) is a family of closely related ubiquitous transcription factors that regulate an extensive array of genes involved in different immune and inflammatory responses. This family is composed of five structurally related proteins including NF-κB1 (p50/p105), NF-κB2 (p52/p100), RelA (p65), c-Rel, and RelB that mediates transcription of target genes by binding to a specific DNA element, κB enhancer, as various hetero- or homo-dimers (8). The NF-κB proteins are usually sequestered in the cytoplasm by a family of inhibitory proteins, including IκB family members and related proteins defined by the presence of ankyrin repeats. Two different signal pathways have been proposed for NF-κB activities, the classical/canonical or non-canonical pathway. Upon stimulation of classical pathway by Toll-like-receptors, or B and T cell receptors, the IκKB subunit phosphorylates and polyubiquitinates, leading to its degradation by the 26S proteasome (8) and translocation of p50 into the nucleus to exert its function as a transcription factor (9).

We have recently identified the pathogenic impact of identified *NFKB1* deleterious variants in our PAD cohort; we have shown that missense mutation in the *NFKB1* causes late-onset PAD by impairing the function of the transcriptionally active p50 (10). We identified further a previously uncharacterized *NFKB1* missense variant in a family with a history of meningococcal meningitis and late-onset hypogammaglobulinemia by next-generation sequencing (NGS) as the only predicted deleterious variant within genes of inborn errors of immunity. Hence in the current work, we mainly focused on the relevance of this NFKB1 missense variant (c.691 C>T, p.R230C) by immunophenotyping, immunoblotting, and ectopic expression assays.

## Materials and Method

### Ethical Aapproval

The institutional medical ethical committee at Hannover Medical School approved the study (ethics approval number: Nr.8875_BO_K_2020). The written consent of all study participants was obtained.

### Isolation of Genomic DNA and Sequencing Methods

Genomic DNA (gDNA) was isolated from peripheral blood of patients and healthy donors with QIAamp Kit (QIAamp DNA Blood Midi Kit; Lot# 16902455*; Qiagen*). Whole exome sequencing (WES) was performed on genomic DNA samples from patients S1 and S2 as described previously (11). Briefly, the concentration and quality of the purified genomic DNA (gDNA) was determined with an Agilent Technologies 2100 Bioanalyzer (Agilent Technologies, Santa Clara, CA, USA). The DNA sequencing library consisted of 100 ng fragmented gDNA and was generated with Agilent SureSelectXT Reagent Kits v5 UTR (70 Mb) according to the manufacturer’s protocols (Illumina, San Diego, CA, USA). Libraries were sequenced on an Illumina HiSeq2500 platform using TruSeq SBS Kit v3-HS (200 cycles, paired end run) with an average of 12.5 × 106 reads per single exome (mean coverage: 50X). The GATK-Pipeline (GenomeAnalysisTK-1.7) was applied for read quality trimming, read alignment to reference (GRCh37/hg19) and quality trimmed variant calling. Variant annotation was performed using Gsvar software. We selected for rare variants with low minor allele frequency (MAF < 0.05). Sanger sequencing was performed by (*Eurofins Genomics*) to validate the identified rare *NFKB1* variant and its co-segregation with disease phenotype in this family using the primers: forward; 5'-GTCTATTCTTGGTGTGCCCC-3' and reverse; 3'-TGCAGCAGACCAAGGAGATG-5'.

### PBMCs Isolation

Whole blood was collected from both patients and healthy control. PBMCs were isolated using the standard centrifugation method. Briefly, whole blood was mixed with PBS in a 1:2 ratio, and the diluted cell suspension was gently layered on the Ficol-plaque separation gradient. Centrifugation was carried out at 1000xg for 20 mins with no break at 21°C. The mononuclear cell layer was carefully removed, transferred into new 50 ml falcon tubes, and washed with PBS. Cells were either stored in 10% DMSO or immediately used.

### Lymphocyte Stimulation Assays

For stimulation experiments, PBMCs were treated with phorbol 12-myristate 13-acetate (PMA; 50 ng/ml) and ionomycin (1 μg/ml) and incubated for 30 min at 37°C and 5% carbon dioxide. After incubation cells were harvested and used immediately for total protein extraction.

For standard T cell proliferation assays, PBMCs were stimulated with phytohemagglutinin (PHA), concanavalin A (ConA), pokeweed mitogen (PWM), purified protein derivative (PPD), interleukin 2 (IL-2) and anti-CD3mAb as described previously (10, 12).

### Cell Culture and Transfection

Human Embryonic Kidney 293 (HEK 293) cells were cultured in Dulbecco’s modified Eagle’s medium (DMEM) supplemented with 10% heat-inactivated fetal bovine serum (FBS), 1% penicillin/streptomycin, and 1% Sodium pyruvate (Invitrogen) at 37°C with 5% CO2. A day before transfection, 5 ×10^5 HEK 293 cells/ well were seeded into a six-well plate and cultured at 37°C. Transfection was carried out using a 2 µg plasmid, each using x-tremegene hp DNA transfection reagent (Merck, 6366244001) following the manufacturer’s protocol utilizing eGFP plasmid expression vector and Opti-Mem as a negative control. Transfected cells were selected two days post-transfection using 400 µg/ m G418 sulfate.

### Cloning

Plasmids encoding either p105 or p50 wild type and mutant with N-terminal GFP tag were cloned into pc.DNA3.1 (+)-N-eGFP (Genescript). Competent *E. coli* (NEB, 5-alpha) were transformed with the vectors constructs, and plasmids were isolated with QIAprep Spin Mini Kit (Qiagen).

### Fluorescence Staining and Confocal Imaging

HEK293 cells that were transiently expressing eGFP alone or N-terminally EGFP-tag p50-wt or p50-R230C mutant were fixed with 4% paraformaldehyde for 20 mins at RT followed by 3X washing with PBS and permeabilized with 0.1% Triton X-100. The nuclei were stained with DAPI. For microscopy analysis, glass coverslips were mounted with a drop of the fluorescence-mounting medium (DAKO). Confocal fluorescence images were taken on an Olympus FV1000 laser-scanning microscope. Images were evaluated and processed with Fiji software.

### Western Blotting and qRT-PCR

Total RNA and proteins were isolated from transfected HEK293 cells or subjects´ and healthy control PBMCs using NE-PER Nuclear and cytoplasmic extraction reagent (Thermo Scientific; lot # TA259812) and Allprep DNA/RNA micro-kit (Qiagen; #80284), respectively. Proteins were separated on 7.5% Mini-Protean TGX (BIO-RAD; #4561024) and transferred onto Invitrogen PVDF membranes (Thermo Scientific). Both p105 and p50 proteins were detected using a primary polyclonal rabbit antibody directed against the N-terminal amino acids (Cell Signaling; #3035). A monoclonal rabbit antibody was used to detect levels of phosphorylated p105 at serine 933 (#4806; *Cell Signaling*). Horseradish-peroxidase-coupled goat anti-rabbit secondary antibody (#6721; Abcam) was used to detect signals *via* enhanced chemiluminescence (SuperSignal West Dura Extended Duration Substrate; Thermo Fischer). For the loading controls rabbit beta actin (#12620; cell signaling) and histone H3 (#12648; cell signaling) directly coupled to horseradish-peroxidase was used. Rabbit antibody against beta-actin directly coupled to horseradish peroxidase was used as a loading control. For quantitative Real-Time polymerase chine reaction (qRT-PCR), 1 µg of total RNA was used for cDNA synthesis. Reverse transcription was performed using High-Capacity cDNA Reverse Transcription Kit (Applied biosynthesis, Lithuania) following the manufacturer’s protocol. No RNA and no reverse transcriptase served as the negative control. Expression levels of NFκB1 and GAPDH transcript were quantified using Taqman Gene Expression Master Mix with custom-made probes were used for the qRT-RT assay. The average changed in threshold cycle values was determined for each sample relative to the endogenous GAPDH levels and compared with the control.

### Statistics 

Statistical analyses were performed using Graph Pad Prism, version 8. One-way analysis of variance (ANOVA) test was used to compare *NFκB1* expression. Densitometry analyses of western blots were performed with ImageJ software (Version 1.52 v), and the graphs were prepared using Microsoft Excel 365.

## Results 

### Identification of *NFKB1* Missense Variants by tNGS

**Figure 1:**
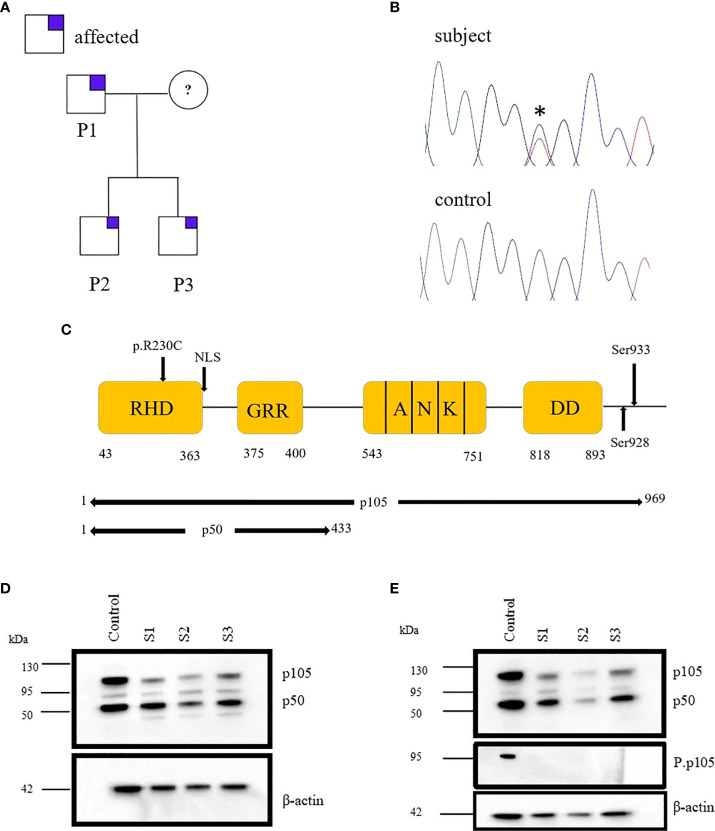
Monoallelic *NFKB1* missense mutation in a family with late-onset antibody deficiency. **(A)** Segregation of NFKB1variant was analyzed by sequencing genomic PCR product and revealed an autosomal-dominant inheritance in families with reduced clinical penetrance. The analysis excluded the mother of patients because of material lack. **(B)** Sanger sequencing of genomic PCR products results in the chromatogram of missense variant and wild type (WT) **(C)** Structure of NFκB protein showing the position of the identified mutation. **(D)** Immunoblotting was performed in PBMCs of subjects (S1, S2, and S3) and healthy control (HC), and the expression of p105/50 was evaluated. The expression of p105 was reduced for all the subjects compared to the control. However, the expression of p50 was reduced in S2. **(E)** PBMCs from HC and S1, S2 and S3 were stimulated with PMA; 50 ng/ml and ionomycin; 1 μg/ml and the expression of p105/50 evaluated. There were no significant changes in the p105/50 expression after stimulation in the subjects; however, p105 phosphorylation at serine 933 was detected in only the HC but not in the subject. Beta-actin was used as a cytoplasmic loading control.

We identified a *NFKB1* deleterious missense variant (691 C>T, p.R230C) in a family with antibody deficiency (**Figure 1A**). This variant was confirmed by Sanger sequencing (**Figure 1B**) and predicted to be deleterious by *in silico* tools.

### Clinical History 

A 23-year-old patient (S2) born to non-consanguineous Caucasian healthy parents of German descent presented to our Immunology outpatient clinic due to a history of meningococcal meningitis with sepsis (Waterhouse–Friderichsen syndrome) at the age of 10 years. He additionally had a parechovirus meningitis at the age of 20 years. Except for the aforementioned two meningitides and the identification of 2-3 isolated boils through the physical examination, his infection record was inconspicuous. This patient’s two years younger brother (S3) also suffered from meningococcal meningitis with sepsis at the age of 17 years, at a different time point than his brother. He additionally reported recurrent bronchitis and one pneumonia. On the bases of immunological investigations S2 were consistent with the diagnosis of a combined immunodeficiency (CID), whereas S3 was diagnosed with common variable immunodeficiency (CVID). Findings of immunological investigations of both brothers are summarized in **Table 1**. Patients' clinical and immunological values are regularly monitored and examined in our clinic. Furthermore, none in the family had a history of severe or recurrent infections.

**Table 1:** Clinical and immunological characteristics of studied subjects with NF-κB1 LOF variant.

	S.1	S.2	S.3	Reference range
**Year of birth**	1974	1998	2000	
**Sex**	male	male	male	
**Infections**	None reported	*Neisseria meningitidis* meningitis and sepsis (Waterhouse-Friderichsen-syndrome); *Parechovirus* meningitis; isolated furuncle	*Neisseria meningitidis* sepsis (Waterhouse-Friderichsen-syndrome); one pneumonia; twice bronchitis within a year	
**Full blood count:**				
WBC (cells/µl)	6100	8000	5700	4800-12000
Lymphocytes (cells/µl)	1200	1160	**940**	1100-4500
Lymphocytes (% WBC)	**16.0**	**14.8**	**16.5**	20-44
Monocytes (% WBC)	8.7	8.8	8.3	2-9.5
Neutrophils (% WBC)	72.9	74	72.2	42-77
**Phenotypic profile of peripheral blood lymphocytes:**				
CD3+ T cells (% lymphocytes)	36.6	50	69	55-83
CD4+ T cells (% lymphocytes)	**45**	**31.6**	**45.4**	55-83
CD8+ T cells (% lymphocytes)	29.3	15.1	18.6	10-39
CD19+ B cells (% lymphocytes)	10.7	**12**	**5**	6-19
γδ T-cell (% T cells)	**27.7**	**15.1**	9.6	<10%
CD3+CD56+ NK cells (% lymphocytes)	**6.9**	22.3	22.3	7-31
**Phenotypic profile of peripheral blood CD4+T cells:**				
naive CD4+ T cells (% of CD4+ T cells)	**46.9**	**74.6**	67.8	(49-72)
memory CD4+ T cells (% CD4+ T cells)	46.5	**17.4**	**14.3**	34 -71
recent thymic emigrant (RTE) T helper cells (% CD4+ T cells)	**20.6**	48.4	55	42-64
**Phenotypic profile of peripheral blood CD8+T cells:**				
early effector memory CD8+ T cells (% of CD8+ T cells)	13.9	5.8	11.5	2.9-16
late effector memory CD8+ T cells (% CD8+ T cells)	9.5	**1.6**	4.8	2.6-58
**Phenotypic profile of peripheral blood CD19+ B cells:**				
Naive B cells (% B cells)	78.9	71.1	70.7	29-93
IgM+ memory B cells (% B cells)	5.8	5.9	7.1	2-25
class-switched B cells (% B cells)	6.9	14.6	14.3	3-23
transitional B cells (% B cells)	**3.7**	1.5	1.2	0.6-4-6
plasmablasts (% B cells)	**5.2**	**7.6**	**5.5**	0.4-3.6
CD21^low^ B cells (% B cells)	5.2	3.7	2.1	1-26
**Immunoglobulins:**				
IgG (g/l)	**6.2**	9.64	**6.95**	7-16
IgA (g/l)	**0.51**	**0.66**	**0.53**	0.7-4
IgM (g/l)	0.5	0.91	0.62	0.4-2.3
Pneumococcal antibody (mg/l)**	n.m.	**110**	**251.7**	39.4-100.5
Tetanus antibody (IU/ml)**	n.m.	1.2	**0.8**	>1.1
**Granulocytes function test:**				
C3c (g/l)	n.m.	1.01	1.4	0.9-1.8
C4 (g/l)	n.m.	0.18	0.14	0.1-0.4
CH50 (U/ml)	n.m.	**>60**	**>60**	31.6-57.6
AP50 (%)	n.m.	**110**	92	60-102

n.m, not measured.

**values after booster vaccination.

Values in bold deviate from reference range.

### Missense Variant 691 C>T, p.R230C Causes p105/p50 Reduction and Impair p50 Nuclear Translocation

**Figure 2:**
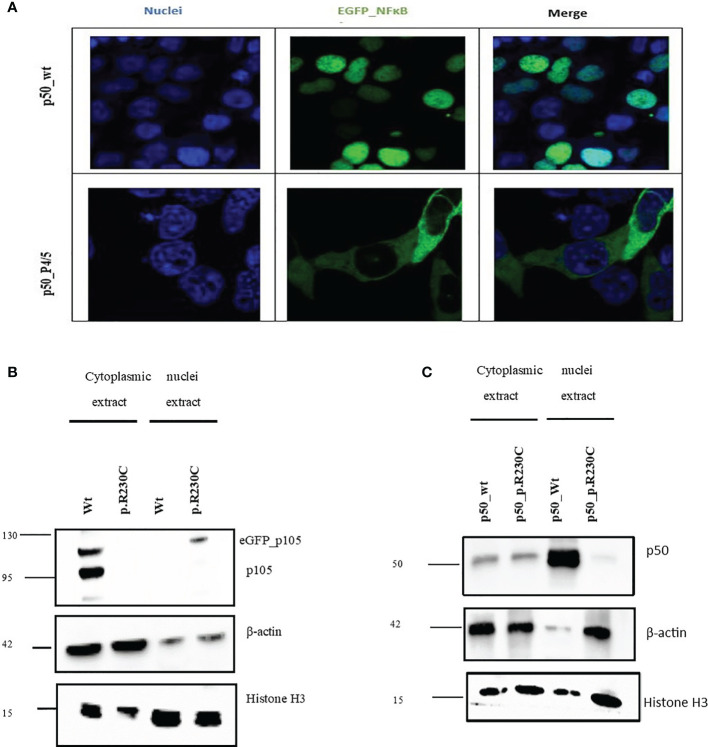
NFkB1 expression in and nuclear translocation in transfected HEK293 cells. Total proteins were extracted from transfected HEK293 cells to determine the expression of p105/50. **(A)** For microscopic analysis of NFkB1-p50 translocation into the nucleus, HEK293 cells transiently expressing the eGFP-p50 wild type and mutant were used. Nuclei were stained with DAPI. The wild-type p50-construct localizes into the nucleus while the mutant p50-construct was retained in the cytoplasm. **(B)** Western blot to analyze the effect of the variant on the expression of p105/50 in the transfected HEK293 cells. The expression in the wild type was prominent in the wild type, while no noticeable expression in the variant was observed. **(C)** p50 expression in HEK293 showed an increase in wild-type and a decrease in the mutant (p. R230C) in the nuclear extract.

Both the two brothers (S2/3) were identified to carry the missense variant 691 C>T, p.R230C in *NFKB1* located on the N-terminal of the RHD (**Figure 1C**). This variant refers to the group of NFKB1 mutations, affecting both the precursor p105 and the mature p50 as we described previously (10). HEK293 cells were transiently transfected with EGFP-fused mutant proteins or EGFP-fused wild-type proteins. Immunoblotting and qRT-PCR were carried out. A reduced mRNA level and a drastically reduced protein level (approximately 92%) were observed for p105/50 (**Figures 2B**; **Supplementary Figure 1**) in transfected HEK293 cells. In transfected HEK293 cells, the EGFP-fused p50-R230C mutant showed an aberrant localization into the nucleus while the wild-type localization was normal (**Figure 2A**). More importantly, the expression of p50 using the EGFP-fused p50-R230C mutant in HEK293 showed that p50 expression in the mutant was reduced compared to the wild type (**Figure 1C**). These results indicate that the functional defect influences both the precursor p105 and mature p50.

PBMCs from mutation carriers (n=3), healthy control (n=1) were stimulated with PMA plus ionomycin and protein were extracted from cytoplasm and nucleus and analyzed by western blotting. Representative results are shown (**Figures 1D, E**). p105 amounts, phosphorylation of p105, and processing to p50 are severely reduced compared to control. The β-actin control confirms equal loading.

## Discussion

### The Pathogenic *NFKB1* c.691 C>T, p.R230C Variant Causes Progressive Late-Onset Antibody Deficiency

This study assayed the pathogenicity of a deleterious missense NFKB1 variant, c.691 C>T, detected in two brothers (S2/3) and their father (S1) from a German family with variable clinical presentations but a shared history of meningococcal meningitis with sepsis and viral meningitis in the different points of their childhood and adolescence. Later, both developed CID and CVID, respectively. The variant is located in the N-terminal part of p105; using the *in vitro* transfection model, we found an impaired nuclear translocation of active p50.

Neisseria meningitidis is a restricted human bacterium and a common nasopharynx colonizer. The bacterium is often harmless but, in rare cases, can cause life-threatening meningitis and sepsis. There is solid evidence for the role of host genetics in predisposition to meningococcal infection. So far, monogenic defects in the terminal component of complement and polymorphism in the CFH/CFHR3 region have been described to be associated with susceptibility to meningococcal meningitis (13). An association between meningococcal meningitis and NFKB1 is not reported yet.

Recently performing the ectopic expression in HEK293T cells, we have shown that frameshift mutations in N terminal “p50 half” of p105 lead to seriously truncated proteins that lack the nuclear localization sequence (NLS) and consequently cause rapid proteasomal degradation (10). The overall expression levels of both p105 and p50 were decreased by about ~50% compared to healthy controls, as previously reported for several NFKB1 haploinsufficiency variants (6, 7, 10). Almost all patients with alteration in NFKB1 causing p50 haploinsufficiency presented with diverse clinical manifestations, mainly autoimmunity, lymphoproliferative disorders, splenomegaly, CMV infections, and malignancies besides antibody deficiency (14). Our reported patient with truncating mutation presented the most severe phenotype; his sons carry the same mutation; however, one displayed mild IgG while the other showed autoimmune thyroiditis and asthma bronchial (10). Therefore, both are under closer follow-up because IgG deficiencies can develop into CVID over time, and the age-dependent exhibition of the NF-κB1-related phenotype has been reported (14).

Deleterious mutations in the N terminal part of p105, including frameshift and truncations, have been shown to cause (haplo) insufficiency, whereas examining the pathogenic relevance of a vast number of *NFKB1* missense variants in patients with immunodeficiency remained scarce. The recorded variant p.H67R reduced nuclear entry of p50 and showed decreased transcriptional activity in a luciferase reporter assay (15). The three rare substitutions (p.I281M; p.V98D, p.I87S) identified in sporadic CVID have been shown to reduce p50 levels and affect protein stability. We have recently demonstrated that the rare p.R157P variant reduced p105/p50 expression in the patient’s derived cells, whereas the EGFP-fused mutant p50 revealed an aberrant intranuclear pattern (10). Most recently, the characterization of p.Y350C substitution in transfected HEK293T cells has shown decreased p105 expression, indicating an accelerated decay, although forced expression of mutant p50 affected nuclear translocation (16). Recently, Li et al. evaluated the functional impact of 365 NFKB1 variants utilizing a reporter assay and showed that deleteriousness of monoallelic variants in NFKB1 lies on haploinsufficiency. Characterized *NFKB1* variants included missense LOF or hypomorphic variants, which all – similar to the variant presented in the present work – were localized at the RHD domain of p105/p50 (17).

In the present study, we investigated the impact of variant c.691 C>T, p. R230C by overexpression assay of EGFP-fused NFκB1 proteins. HEK293 cells were transiently transfected with EGFP-fused mutant proteins or EGFP-fused wild-type proteins. Confocal microscopy analysis also showed that p50 was sequestered in the cytoplasm, whereas wild-type localization was in the nucleus (**Figure 2A**). Immunoblotting revealed reduced expression of mutant p105 compared to the wild type (**Figure 2B**) and a drastic diminish of p50 mutant in the nucleus compared to the wild-type counterpart (**Figure 2C**), indicating the functional defect affects both and predominately the p50 localization into the nucleus.

The assessment of NFκB1 protein levels in the PBMCs of the father (S1) and the two brothers (S2/3) was investigated. We observed a reduction in p105/50 in all the missense variants carriers compared to the control (**Figure 1D**). More importantly, the expression of p50 was substantially reduced for S2. Although the patients' father carries the same mutations with a reduced p105 expression, he is clinically unaffected. This observation is consistent with previous studies that observed a reduction in p105/50 expression in clinically and non-clinically affected family members (18).

Indeed, the diverse clinical phenotypes are seen in these brothers harbouring the same mutation refer to the incomplete penetrance nature of the disease and might be explained by additional factors such as environmental and or epigenetic alterations (19) that necessitate further investigations. With the coming of NGS in several independent studies, *NFKB1* mutations have been described as the most frequent monoallelic genetic cause of PAD with variable clinical phenotype, even within the same affected family (20). In addition, recent cohort studies on patients with *NFKB1* variants have shown that infections, lymphoproliferative disorders, autoimmune diseases, and malignancies are the most common and age-dependent manifestations in these patients (14). Besides its role in regulating B-cell activities such as differentiation and proliferation, NF-kB1 is also crucial for T-cell activation, antigen presentation, and regulation of tissue-specific autoimmunity (21). Thus reduction in p105/p50 expression may play a role in autoimmunity, lymphadenopathy, and splenomegaly as observed in mice lacking p105 expression (22). Our patients currently present milder clinical phenotypes with hypogammaglobulinemia, moderate lymphopenia, and reduced CD4+T cells. Since family members, carrying deleterious NFKB1 variants with moderate phenotype are at high risk for autoimmunity and malignancy, a closer follow-up for early initiation of IgG substitution therapy to prevent complications is strongly recommended.

## Conclusion

Pathogenic variants within RHD impairs the p50 nuclear translocation and, therefore, might diminish the functions of p50, resulting in progressive antibody deficiency and suggests the need for closer monitoring and counselling of mutation carriers. This study is a further crucial extension of our knowledge in the NF-κB1-related phenotype.

## Data Availability Statement

The original contributions presented in the study are publicly available. This data can be found here: https://www.ncbi.nlm.nih.gov/bioproject/PRJNA788443.

## Ethics Statement

The studies involving human participants were reviewed and approved by the institutional medical ethical committee at Hannover Medical School approved the study (ethics approval number: Nr.8875_BO_K_2020). The written consent of all study participants was obtained. The patients/participants provided their written informed consent to participate in this study.

## Author Contributions

Research design, FA and RS. Sample collection, FA, GeS, and MA. WES, GuS. NGS data analysis, FA. Performance of functional experiments and data analysis, MA. Writing and contributing to writing of the manuscript FA, M.A, and all authors. All authors contributed to the article and approved the submitted version.

## Funding

This project was funded by the Deutsche Forschungsgemeinschaft (DFG, German Research Foundation) under Germany’s Excellence Strategy—EXC 2155 'RESIST'—Project ID 39087428 and the German network for multi-organ autoimmune diseases GAIN_ 01GM1910A. GeS receives funding from the Young Academy Clinician/Scientist program of Hannover Medical School, Germany and the Rosemarie-Germscheid foundation. All authors and this project are supported by the German Center for Infection Research (DZIF TTU 07.801).

## Conflict of Interest

The authors declare that the research was conducted in the absence of any commercial or financial relationships that could be construed as a potential conflict of interest.

## Publisher’s Note

All claims expressed in this article are solely those of the authors and do not necessarily represent those of their affiliated organizations, or those of the publisher, the editors and the reviewers. Any product that may be evaluated in this article, or claim that may be made by its manufacturer, is not guaranteed or endorsed by the publisher.

## Supplementary Material

Supplementary Figure 1 Total RNA was extracted from transfected HEK293 cells to determine the expression of p105 at the mRNA level. (A) Quantitative-Real time PCR showed a reduction in the transcript levels of p105 in the mutant compared to the wild type. Delta delta Ct was then determined by subtracting ΔCt (variant) from ΔCt (wild type). Fold change in expression was calculated using 2-ΔΔCt.

Supplementary Figure 2 Densitometry analysis. Densitometry analysis was performed for p105 and p50 expression in the transfected HEK293 cells and PBMCs. **(A)** Expression of p105 in HEK293 cells transfected with wild type NFκB1 and mutant (p.R230C). **(B)** Expression of p50 in HEK293 cells transfected with wild type NFκB1 and mutant (p.R230C) showed a reduction in the p50 by immunoblotting. Both p105 and p50 were drastically reduced in the mutant compared to the wild type. **(C)** Expression of p105/50 in the PBMCs of healthy control (HC) and mutation carriers (S1-S3). Densitometry analysis revealed lowered expression in all subjects compared to the control. **(D)** PBMCs from HC and S1, S2 and S3 were stimulated with PMA; 50 ng/ml and ionomycin;1 μg/ml and the expression of p105/50 examined. There was no significant changes in the p105/50 expression after stimulation.
