# Corrigendum: Vulnerability to meningococcal disease in immunodeficiency due to a novel pathogenic missense variant in *NFKB1*

**DOI:** 10.3389/fimmu.2023.1212029

**Published:** 2023-05-10

**Authors:** Manfred Anim, Georgios Sogkas, Gunnar Schmidt, Natalia Dubrowinskaja, Torsten Witte, Reinhold Ernst Schmidt, Faranaz Atschekzei

**Affiliations:** ^1^ Department of Rheumatology and Immunology, Hannover Medical School, Hannover, Germany; ^2^ Hannover Biomedical Research School (HBRS), Hannover Medical School, Hanover, Germany; ^3^ RESIST - Cluster of Excellence 2155 to Hanover Medical School, Satellite Center Freiburg, Hanover, Germany; ^4^ Department of Human Genetics, Hannover Medical School, Hannover, Germany

**Keywords:** common variable immune deficiency (CVID), *NFKB1*, Nfkb1 (p50), hypogammaglobulinemia, primary antibody deficiency (PAD)

In the published article, there was an error in **Figure 1** as published. We recognized that the loading control (β-actin) in **Figure 1E** is identical with **1D** and has to be replaced with the correct loading control.

**Figure 1:**
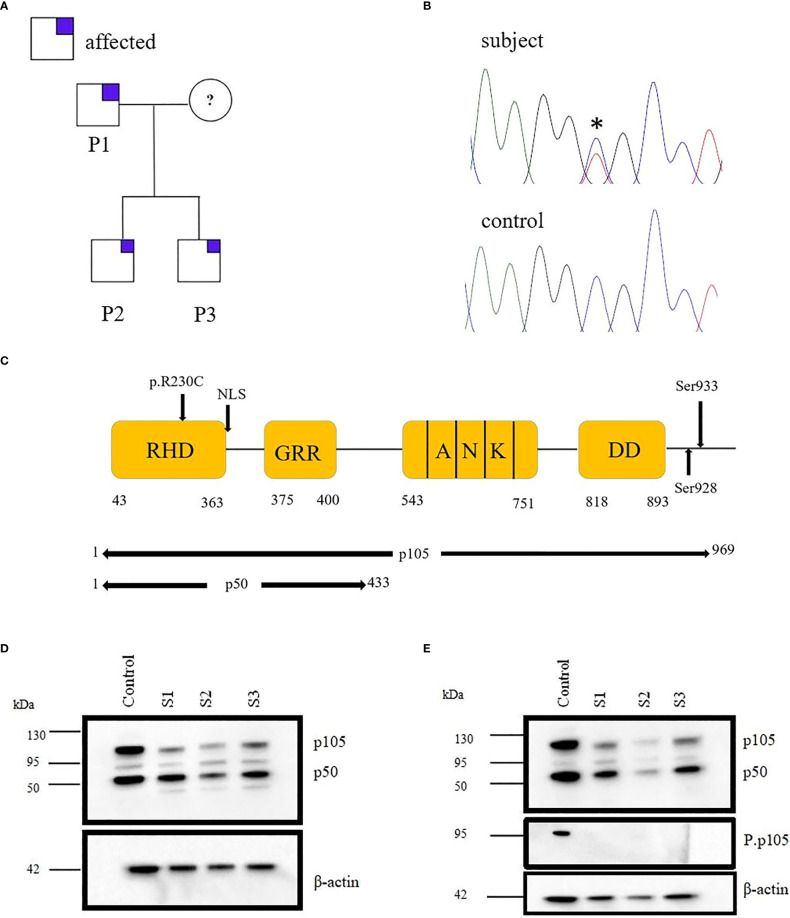
Monoallelic *NFKB1* missense mutation in a family with late-onset antibody deficiency. **(A)** Segregation of NFKB1variant was analyzed by sequencing genomic PCR product and revealed an autosomal-dominant inheritance in families with reduced clinical penetrance. The analysis excluded the mother of patients because of material lack. **(B)** Sanger sequencing of genomic PCR products results in the chromatogram of missense variant and wild type (WT) **(C)** Structure of NFκB protein showing the position of the identified mutation. **(D)** Immunoblotting was performed in PBMCs of subjects (S1, S2, and S3) and healthy control (HC), and the expression of p105/50 was evaluated. The expression of p105 was reduced for all the subjects compared to the control. However, the expression of p50 was reduced in S2. **(E)** PBMCs from HC and S1, S2 and S3 were stimulated with PMA; 50 ng/ml and ionomycin; 1 μg/ml and the expression of p105/50 evaluated. There were no significant changes in the p105/50 expression after stimulation in the subjects; however, p105 phosphorylation at serine 933 was detected in only the HC but not in the subject. Beta-actin was used as a cytoplasmic loading control.

The corrected **Figure 1** and its caption [Monoallelic *NFKB1* missense mutation in a family with late-onset antibody deficiency. (A) Segregation of NFKB1variant was analyzed by sequencing genomic PCR product and revealed an autosomal-dominant inheritance in families with reduced clinical penetrance. The analysis excluded the mother of patients because of material lack. (B) Sanger sequencing of genomic PCR products results in the chromatogram of missense variant and wild type (WT) (C) Structure of NFκB protein showing the position of the identified mutation. (D) Immunoblotting was performed in PBMCs of subjects (S1, S2, and S3) and healthy control (HC), and the expression of p105/50 was evaluated. The expression of p105 was reduced for all the subjects compared to the control. However, the expression of p50 was reduced in S2. (E) PBMCs from HC and S1, S2 and S3 were stimulated with PMA; 50 ng/ml and ionomycin; 1 μg/ml and the expression of p105/50 evaluated. There were no significant changes in the p105/50 expression after stimulation in the subjects; however, p105 phosphorylation at serine 933 was detected in only the HC but not in the subject. Beta-actin was used as a cytoplasmic loading control.] appear below.

The authors apologize for this error and state that this does not change the scientific conclusions of the article in any way. The original article has been updated.

