# Supplementary material for: Vulnerability to Meningococcal Disease in Immunodeficiency Due to a Novel Pathogenic Missense Variant in NFKB1
Source: Front Immunol. 2021 Dec 24;12:767188. doi: 10.3389/fimmu.2021.767188 (PMC8738076; doi:10.3389/fimmu.2021.767188)
Supplement: Supplementary Figure 1 — Total RNA was extracted from transfected HEK293 cells to determine the expression of p105 at the mRNA level. (A) Quantitative-Real time PCR showed a reduction in the transcript levels of p105 in the mutant compared to the wild type. Delta delta Ct was then determined by subtracting ΔCt (variant) from ΔCt (wild type). Fold change in expression was calculated using 2-ΔΔCt. [file DataSheet_1.docx]

Supplementary Material

**
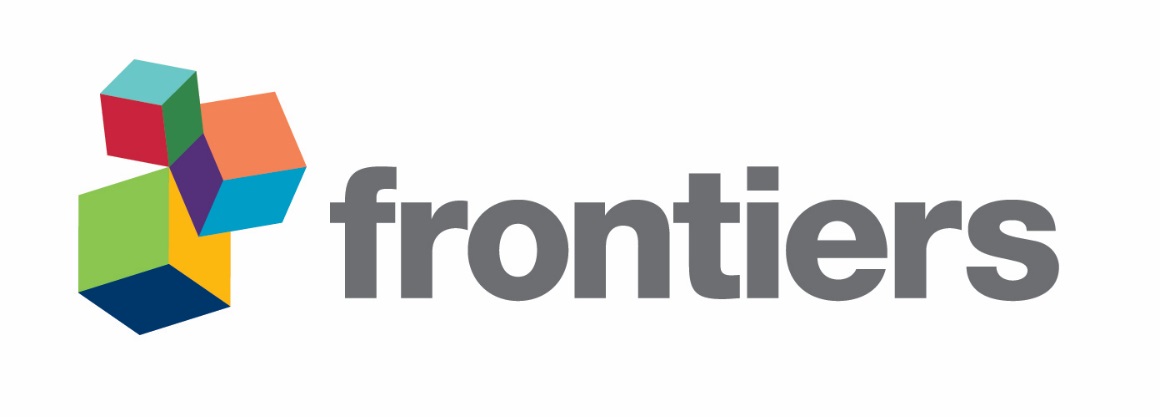
**

**Supplementary data**

**Supplementary Figure 1 (S1)**

**Supplementary Figure 2 (S2)**

b

a

|  |  |  |
| --- | --- | --- |
| c |  | d |
